# Retrospective correlation analysis of plasma Immunoglobulin G and clinical performance in CIDP

**DOI:** 10.7717/peerj.6969

**Published:** 2019-05-16

**Authors:** Lars Kjøbsted Markvardsen, Stine Bruun-Sørensen, Ingelise Christiansen, Henning Andersen

**Affiliations:** 1 Department of Neurology, Aarhus University Hospital, Aarhus, Denmark; 2 Department of Neurology, Rigshospitalet, Copenhagen, Denmark

**Keywords:** Muscle strength, CIDP, Plasma immunoglobulin G, IVIG, SCIG

## Abstract

**Background:**

Chronic inflammatory demyelinating polyneuropathy (CIDP) can be successfully treated with immunoglobulin either intravenously (IVIG) or subcutaneously (SCIG). Measurement of plasma immunoglobulin G levels (P-IgG) and its correlation to clinical improvement has shown conflicting results. This study aims to clarify whether changes in P-IgG are related to clinical development in patients with CIDP treated with IVIG or SCIG.

**Methods:**

Patients from five previous studies treated with either IVIG or SCIG with evaluation at baseline and re-evaluation after two or 10/12 weeks, respectively were included. At evaluation and re-evaluation, the following tests were done: combined isokinetic muscle strength (cIKS), grip strength, 9-hole-peg test (9-HPT), 40-meter-walk test (40-MWT), clinical examination of muscle strength score by the Medical Research Council (MRC) and measurement of plasma immunoglobulin G (P-IgG).

**Results:**

Fifty-five patients were included in the IVIG group and 41 in the SCIG group. There was no correlation between the changes in P-IgG and cIKS in neither the IVIG group (*r* = 0.137, *p* = 0.32) nor the SCIG group (*r* =  − 0.048, *p* = 0.77). Similarly, no correlations could be demonstrated between P-IgG and grip strength, 9-HPT, 40-MWT or MRC.

**Conclusions:**

In patients with CIDP receiving SCIG or IVIG, changes in P-IgG during treatment did not correlate with changes in muscle strength or other motor performance skills.

## Introduction

For decades, first line treatment of chronic inflammatory demyelinating polyneuropathy (CIDP) has been intravenously administered immunoglobulin (Elovaara et al., 2008). In recent years, subcutaneous administration of immunoglobulin (SCIG) has been increasingly used as an evidence based alternative to intravenous administration (IVIG) (Racosta, Sposato & Kimpinski, 2016).

Measurement of plasma immunoglobulin G (P-IgG) can be used in patients with immunodeficiency to monitor treatment with IVIG or SCIG (McCormack, 2012). However, the clinical relevance of P-IgG in CIDP remains unknown.

Few studies have evaluated whether a correlation between P-IgG and clinical improvement can be demonstrated; so far, only few and conflicting results have been reported.

In a study of SCIG versus placebo in patients with CIDP responding to IVIG, we could not find any relation between changes in muscle strength and changes in P-IgG (Markvardsen et al., 2013). In treatment-naïve patients with CIDP we found that the baseline level of P-IgG before initiation of treatment was inversely correlated to improvement of muscle strength (Markvardsen et al., 2017).

Finally, a positive correlation between ΔP-IgG and the clinical outcome has been demonstrated in Guillain-Barré syndrome (Kuitwaard et al., 2009).

The aim of this study was to evaluate the relationship between motor performance and changes in P-IgG in a large cohort of patients with CIDP treated with IVIG or SCIG.

## Methods

### Study design

In this analysis, data were extracted from five previous clinical trials evaluating the effect of IVIG and/or SCIG treatment in CIDP and included the following trials: (i) SCIG-placebo (Markvardsen et al., 2013), (ii) *De novo* (Markvardsen et al., 2017), (iii) SCIG-follow up (Markvardsen et al., 2014), (iv) IVIG-SCIG (Christiansen, Markvardsen & Jakobsen, 2018) and (v) SCIG-SCIG (clinicaltrials.gov: NCT02111590). In these studies, all patients fulfilled the European Federation of Neurological Societies/Peripheral Nerve Society (EFNS/PNS) criteria for CIDP. Exclusion criteria were age <18 years and other forms of peripheral neuropathy. The inclusion criteria for this study was that all patients had undergone the same evaluations at both the evaluation and re-evaluation test. Patients were excluded if treatment was not maintained for the entire follow-up period of either two or 10/12 weeks, if they were treated with placebo, or if they were treatment-naïve. Furthermore, patients participating in several of the included studies were only represented with data from the first study they participated in. All patients responded to immunoglobulin.

For all patients on SCIG treatment, evaluation and re-evaluation were performed with an interval of 10 or 12 weeks. For some patients in the SCIG group, the week 0 evaluation occurred prior to the first SCIG treatment two weeks after the latest IVIG infusion (IVIG-SCIG and SCIG-placebo). In other patients, the week 0 evaluation was performed after several months of SCIG treatment due to enrollment (SCIG-follow-up and SCIG-SCIG). The time point for injection of SCIG was not standardized to the time point for blood sampling.

For all IVIG treated patients, evaluation and re-evaluation were performed with an interval of two weeks. The baseline evaluation including blood sampling was made before the IVIG infusion and re-evaluation was made two weeks after the first day of the IVIG infusion. Duration of IVIG infusion ranged from two to five days.

### Endpoints

The primary efficacy parameter was change in muscle strength determined by isokinetic dynamometry. Secondary parameters were changes in grip strength (GS), the 9 Hole-peg-test (9-HPT), the 40 meter-walk-test (40-MWT) and the clinical evaluation of muscle strength score by the Medical Research Council (MRC).

### Measurements

At all evaluations, muscle strength was evaluated by isokinetic dynamometry (Biodex System Pro 3 or 4®; Biodex Medical Systems Inc., Shirley, NY, USA) in four pre-selected and mostly impaired muscle groups. The test procedures are described elsewhere (Harbo, Brincks & Andersen, 2012). The value used for the analyses (combined isokinetic muscle strength, cIKS) is the sum of the four muscle groups evaluated.

Grip strength was measured three times for each hand using a hand-held dynamometer (JAMAR®; Lafayette Instrument Inc., Lafayette, IN, USA) and the average value was used in the further analyses.

For the 9-hole-peg test (9-HPT) and the 40-meter walk test (40-MWT), the mean value of a double determination was used. For the 9-HPT, an average for both hands tested was used for analysis.

Finally, a clinical evaluation of muscle strength score was assessed by the Medical Research Council (MRC) (Merkies et al., 2003).

### Statistics

The various parameters of muscle function were tested for Gaussian data distribution and in case this was not fulfilled, a logarithmic transformation or a non-parametric analysis was done.

A correlation analysis was performed by calculation of Pearson’s correlation coefficient and the interpretation was done by evaluating the strength of correlation using the following: <0.25 = no/little correlation; 0.25–0.50 = fair correlation; 0.51–0.75 = moderate correlation; >0.75 = good/excellent correlation (Portney & Watkins, 2009). A level of *p* < 0.05 was considered significant.

## Results

**Figure 1:**
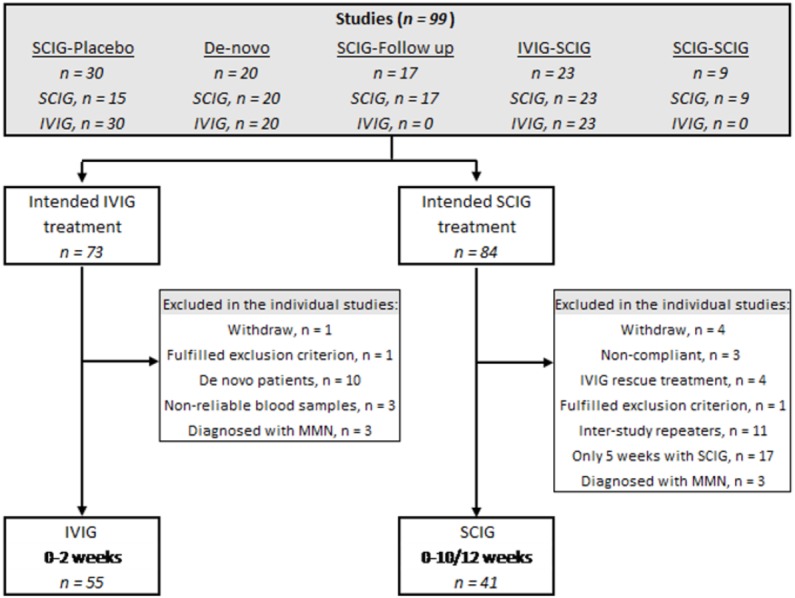
Flowchart of patients eligible for inclusion. The upper box mention the five studies from which the patients are recruited. IVIG, intravenous immunoglobulin; SCIG, subcutaneous immunoglobulin.

Of the 99 patients included in the five studies, 55 patients were eligible for inclusion in the IVIG group and 41 patients for the SCIG group (Fig. 1).

Thirty patients were enrolled in the ‘SCIG-placebo’ study (Markvardsen et al., 2013). One patient withdrew, and 29 patients initially received IVIG and switched to SCIG or placebo. All 29 patients were included in the IVIG-group. Fifteen patients were randomized to placebo and therefore excluded from this study whereas 14 patients were randomized to SCIG. As two patients withdrew their consent, 12 patients were included in this study.

The ‘*De-novo*’ study included 20 patients who were treatment-naïve to immunoglobulin. As 10 patients received IVIG as their first treatment, they were excluded in this study since all patients included from the other studies are known immunoglobulin responders. None of the patients were included in the SCIG group because they were only treated with SCIG for five weeks (Markvardsen et al., 2017).

The ‘SCIG-follow up’ study (Markvardsen et al., 2014) includes patients from the ‘SCIG-placebo’ study (Markvardsen et al., 2013). Seventeen patients were included and as six of them were initial randomized to placebo treatment in the previous trial, they were eligible for inclusion in this study, because the remaining 11 patients were already included from the ‘SCIG-placebo’ study.

In the ‘IVIG-SCIG’ study, 23 patients were switched from regular IVIG to weekly SCIG treatment (Christiansen, Markvardsen & Jakobsen, 2018). Three patients had multifocal motor neuropathy (MMN) and were thus excluded. Twenty patients with CIDP underwent examinations while on IVIG and 14 patients completed the SCIG period of 12 weeks. As three patients were outliers because of non-reliable levels of P-IgG according to IVIG infusion, they were excluded from the IVIG group leaving 17 patients for inclusion.

Finally, in the ‘SCIG-SCIG’ study patients were switched from one SCIG preparation to another clinicaltrials.gov: NCT02111590. Nine patients were eligible for inclusion with two examinations (week 0 and 10).

Twenty-six patients were included in both the IVIG and SCIG group and thereby excluded from one of them. Based on test of outliers, four patients were identified with extreme values in some measurements (40-MWT and/or 9-HPT) and thus excluded in the analyses in this study. Three patients were excluded from the IVIG group because they were outliers in P-IgG levels and due to a negative 1P-IgG when comparing follow-up to baseline.

Table 1 presents the baseline characteristics.

**Table 1:** Baseline characteristics.

	SCIG	IVIG
	*n*= 41	*n*= 55
Gender (M/F)	29/12	40/15
Age (years)	59 (31–76)	59 (36–80)
Body weight (kg)	85 (63–150)	83 (58–122)
Dose of immunoglobulin (g/week)	25 (6–60)	25 (6–48)^a^
Dose of immunoglobulin (g/kg/week)	0.29 (0.08–0.51)	0.32 (0.08–0.51)^a^

**Notes.**

Values are median and (range).

SCIG subcutaneous immunoglobulin IVIG intravenous immunoglobulin

a Patients from IVIG-*de novo* (*n* = 9) are not included due to unknown definite interval of IVIG.

**Figure 2:**
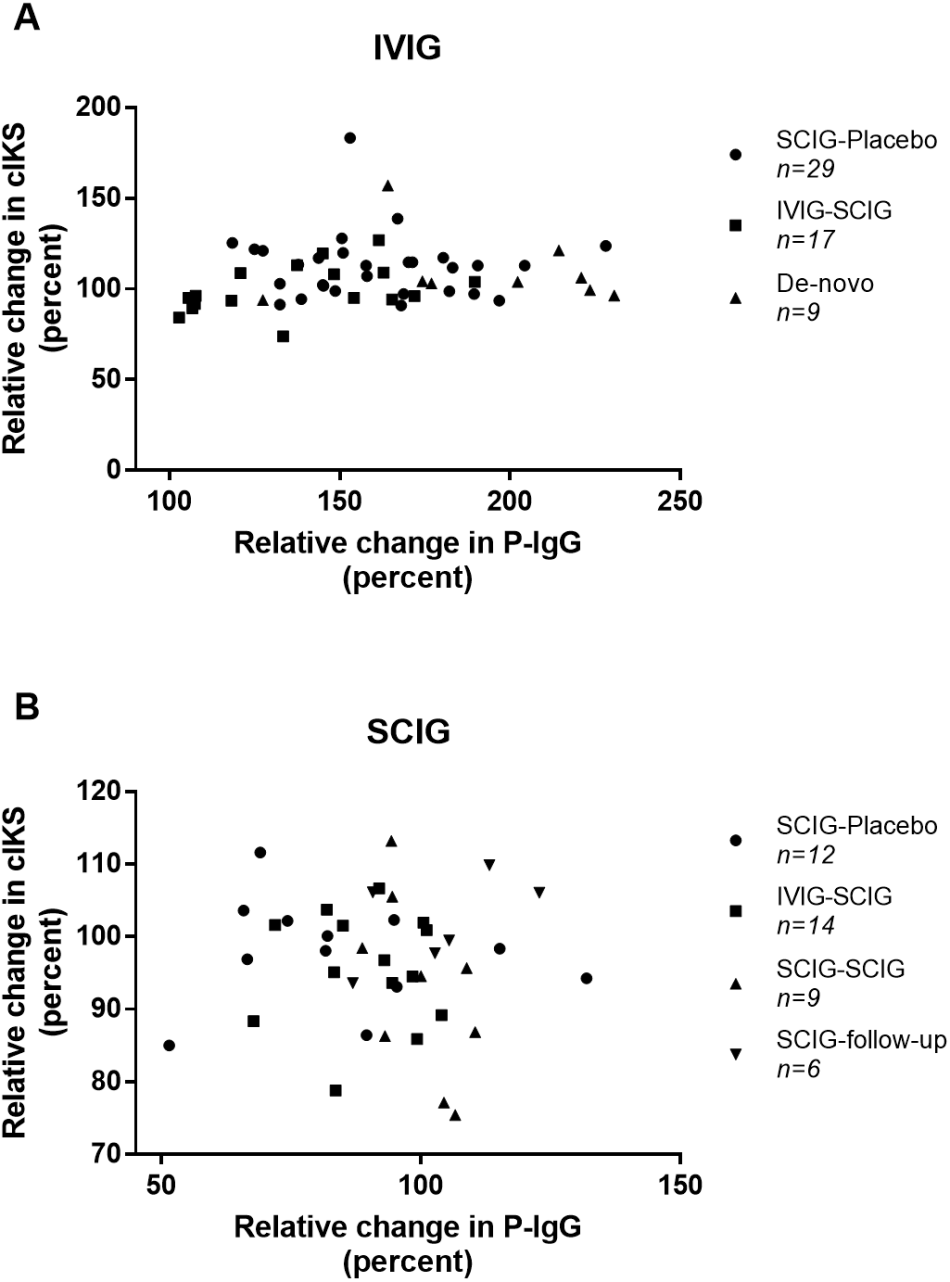
Scatter plot of the correlations between combined isokinetic muscle strength (cIKS) and plasma Immunoglobulin G level (P-IgG). (A) Correlations between cIKS and P-IgG during treatment with IVIG. (B) Correlations between cIKS and P-IgG during treatment with SCIG. Symbols indicating from which study the data come.

We found no correlation between the relative changes in P-IgG and cIKS in neither the IVIG or the SCIG group (*r* = 0.137, *n* = 55, *p* = 0.32 versus *r* =  − 0.048, *n* = 41, *p* = 0.77) (Fig. 2). Correlation analyses in each individual study did not show any correlation (Table S1). The coefficient of variation for cIKS was higher during IVIG than SCIG (16.3% versus 9.3%) but comparable for P-IgG (20.8% versus 17.6%).

Furthermore, no correlation was found when comparing the relative ΔP-IgG to the relative change in any of the secondary parameters (Table 2).

When comparing the absolute baseline P-IgG to ΔP-IgG due to IVIG treatment, we found a negative correlation (*r* =  − 0.318, *n* = 55, *p* = 0.018). Comparing the absolute baseline P-IgG to ΔcIKS, no correlation was found in the IVIG group (*r* =  − 0.198, *n* = 55, *p* = 0.147).

Evaluation of the changes in the measured parameters between the two evaluations showed that all parameters were significantly increased during IVIG treatment, whereas only as small decrease in cIKS and P-IgG was demonstrated during SCIG treatment (Table 3).

## Discussion

This study could not demonstrate any correlation between the relative change in P-IgG and the relative change in cIKS following SCIG or IVIG in patients with CIDP.

In a previous study, we could not detect any relation between plasma IgG and clinical outcome (Markvardsen et al., 2013). In treatment-naïve patients with CIDP treated with IVIG and SCIG, we found an inverse correlation between the baseline level of P-IgG and the increase in cIKS (Markvardsen et al., 2017). This finding could not be confirmed in the present study (*r* =  − 0.198). However, in the present study the patients included were all treated with IVIG as maintenance therapy before inclusion.

**Table 2:** Pearson’s correlation coefficient for the relationship between the relative ΔP-IgG and the relative changes of the effect parameters.

	*SCIG*		*IVIG*	
	*r*	*p*-value	*r*	*p*-value
Grip strength	0.077	0.634	0.018	0.897
9-hole-peg-test	0.126	0.457	−0.128	0.364
40-meter-walking-test	−0.005	0.976	−0.110	0.437
MRC score	−0.089	0.582	0.152	0.268

**Notes.**

*r* Pearson’s correlation coefficient SCIG subcutaneous immunoglobulin IVIG intravenous immunoglobulin MRC Medical Research Council

**Table 3:** Changes in primary and secondary parameters between evaluation and re-evaluation during IVIG or SCIG treatment.

Evaluations *n*=*IVIG/SCIG*	Difference (percent) IVIG	Difference (percent) SCIG
Combined IKS (cIKS)	8.2 (3.4–13.0)*	−3.5 (−6.3 to −0.7)*
*n* = 55∕41		
Grip strength	14.0 (0.7–27.2)*	1.6 (−4.1 to 7.2)
*n* = 55∕41		
9-hole-peg-test	−7.5 (−8.5 to −6.5)*	−1.2 (−3.6 to 1.1)
*n* = 52∕37		
40-meter-walking-test	−8.3 (−12.6 to −4.0)*	−0.6 (−2.9 to 1.7)
*n* = 50∕37		
MRC score	1.7 (0.7–2.8)*	0.8 (−0.3 to 1.8)
*n* = 55∕41		
P-IgG	58.8 (49.9–67.8)*	−7.4 (−12.6 to −2.3)*
*n* = 55∕41		

**Notes.**

* *p* < 0.01.

Rajabally, Wong & Kearney (2013) demonstrated a correlation in IgG1 and IgG2 in 15 patients with CIDP treated with one IVIG infusion. In that study, all patients were regularly treated with immunoglobulin infusions prior to the IgG measurements with a range from two to 10 weeks between IVIG infusions . Moreover, they did not try to correlate the change in IgG to clinical parameters.

In patients with Guillain-Barré syndrome (GBS), Kuitwaard et al. studied the relation between P-IgG and clinical outcome with determination of P-IgG before initiation of IVIG two weeks later. They found that a low ΔIgG was associated with a poorer outcome in patients with GBS (Kuitwaard et al., 2009). In line with our findings in CIDP, the association observed was in treatment-naïve patients after their first immunoglobulin treatment. Based on this, a correlation between the baseline P-IgG and the ΔcIKS may only exist in treatment-naïve patients.

Although a correlation between P-IgG and muscle strength could not be demonstrated, we found a significant inverse correlation between the baseline P-IgG and the absolute ΔP-IgG. However, as the time of the previous IVIG treatment prior to baseline was unknown, it is not possible to apply the findings to daily clinical practice.

Studies have shown that SCIG preserves muscle strength, improves the level of disability and quality of life in patients with CIDP (Cocito et al., 2014; Cocito et al., 2015; Van Schaik et al., 2018). Due to the pharmacokinetics of SCIG, a more stable level with less fluctuation can be obtained by SCIG compared to IVIG (Cocito et al., 2016; Eftimov et al., 2009). In IVIG treated patients, P-IgG increased by 58%, which is comparable to previous studies (81%) (Cocito et al., 2016).

It is the clinical experience that fluctuation in muscle strength is lower during SCIG than IVIG because of a more frequent administration with continuous release of immunoglobulin from the subcutaneous tissue. However, this has not been demonstrated so far. Previously, we have been able to demonstrate a significantly lower variation on the 40-MWT and 9-HPT (Christiansen, Markvardsen & Jakobsen, 2018). Despite the lack of correlation between P-IgG and cIKS, we found that for IVIG both P-IgG and cIKS increased (58% versus 8%), and for SCIG they decreased (−7.4% versus −8%). Moreover, measurements of grip strength, MRC, 9-HPT and 40-MWT indicate that fluctuations are fewer during SCIG treatment as all were unchanged, whereas they all changed significantly during IVIG (Table 3). Thus, the interval between evaluation and re-evaluation is different in the two groups.

The use of disability scores (e.g., INCAT overall disability sum score (ODSS)) is a widely used parameter to detect significant improvements in CIDP. In the studies included, ODSS was obtained, but unfortunately not at all time points. Therefore, these scores do not correspond to the measurement of P-IgG. For a subgroup of IVIG treated (*n* = 20) and SCIG treated patients (*n* = 15), ODSS was obtained at the same time point as P-IgG and in this data subset we found no correlation between the two parameters. This finding is not unexpected as the ODSS is not sensitive to detect small clinical changes.

Limitations to our study is that the time points for the last injection of SCIG and the intervals between IVIG infusions were not standardized to the measurement of muscle strength and functional level. At the re-evaluation, the patients could have taken their SCIG hours or days prior. Although, the P-IgG levels on SCIG treatment is quite stable without major fluctuations compared to IVIG (Cocito et al., 2016), it cannot be ruled out that the time from the last SCIG injection to the evaluation may have influenced the P-IgG level. In IVIG treated patients, the baseline level also fluctuated as the interval of IVIG treatment prior to the different studies ranged from three to 10 weeks; this is comparable to the study by Rajabally, Wong & Kearney (2013). Different infusion intervals clearly affect the measurement of baseline IgG as the serum half-life of IgG is two to three weeks (Lunemann, Nimmerjahn & Dalakas, 2015).

Various conditions could affect the P-IgG level. A high level of immunoglobulin could also be an indicator of an active process (e.g., ongoing infection) in the patients at the time of evaluation. The patients were not tested or evaluated considering taking this into consideration; in case of severe infections, immunoglobulin treatment was not initiated.

Different preparations (Subcuvia®, Shire; Gammanorm®, Octapharma; Hizentra®, CSL Behring) of SCIG are used in the included studies but it has been demonstrated that the bio-availability of the different SCIG preparations did not differ (Berger et al., 2013); thus, the choice of SCIG preparation should not affect the P-IgG level.

Studying a more heterogenous cohort of patients with CIDP, for example including patients with clear wearing-off effect due to IVIG, could help understand and interpret the use of P-IgG measurement in CIDP. On the other hand, this has previously been studied without demonstrating any correlation (Markvardsen et al., 2013; Markvardsen et al., 2017), although this could have been due to the low number of patients. In future studies, P-IgG measurement during tapering off the dosage of SCIG and/or IVIG along with clinical evaluation of muscle strength and clinical performance could add new knowledge on how to interpret measurement of P-IgG in CIDP. Moreover, in treatment-naïve patients we suggest reproduction of P-IgG measurements to confirm a possible correlation to clinical evaluation.

Combining data from five previous studies with different study designs is debatable. We have strictly chosen only to include patients that meet the same inclusion and exclusion criteria and in which data were obtained at the same time points to ensure that data are as comparable as possible. Treatment with IVIG and SCIG was administered equally in all of the included studies, and all measurements were conducted using standardized methods. Clearly, the benefit of using pooled data is that it enables analyses of larger numbers of patients, thus strengthening the power and validity of the data and the conclusions.

Further studies need to investigate whether P-IgG is a potential biomarker for monitoring the clinical effect of IVIG or SCIG in CIDP. This could for example be done by biochemical labelling of IgG in the preparations to be able to distinguish this from the patient’s own IgG. In conclusion, no relation was demonstrated between the ΔP-IgG and the ΔIKS (percentage). Therefore, we suggest that neither the absolute nor the relative P-IgG level can be used as a prognostic factor for the effect of muscle strength during maintenance therapy in CIDP. We thus not recommend analyzing P-IgG as a part of monitoring the disease or treatment effect.

##  Supplemental Information

10.7717/peerj.6969/supp-1 Table S1 Correlations between combined isokinetic muscle strength (cIKS) and plasma Immunoglobulin G (P-IgG) in the individual studies included in the analysis Correlation analyses were made with Spearman correlation coefficient in six of seven comparisons.
For IVIG (IVIG-SCIG study) Pearson correlation coefficient was used due to Gaussian distribution.

10.7717/peerj.6969/supp-2 Data S1 Raw data presenting individual data for each participant

## Additional Information and Declarations

### Data Availability

The following information was supplied regarding data availability: The raw measurements are available as Supplementary File.
