# Supplementary material for: Retrospective correlation analysis of plasma Immunoglobulin G and clinical performance in CIDP
Source: PeerJ. 2019 May 16;7:e6969. doi: 10.7717/peerj.6969 (PMC6526015; doi:10.7717/peerj.6969)
Supplement: Table S1 — Correlation analyses were made with Spearman correlation coefficient in six of seven comparisons. For IVIG (IVIG-SCIG study) Pearson correlation coefficient was used due to Gaussian distribution. [file peerj-07-6969-s001.docx]

**Supplementary table 1**

Correlations between combined isokinetic muscle strength (cIKS) and plasma Immunoglobulin G (P-IgG) in the individual studies included in the analysis

|  |  |  |  |
| --- | --- | --- | --- |
|  | n | r | P-value |
| **IVIG** |  |  |  |
| SCIG-Placebo | 29 | -0.07 | 0.73 |
| IVIG-SCIG | 17 | 0.42 | 0.10 |
| De-novo | 9 | -0.16 | 0.68 |
|  |  |  |  |
| **SCIG** |  |  |  |
| SCIG-placebo | 12 | -0.08 | 0.81 |
| IVIG-SCIG | 14 | 0.019 | 0.95 |
| SCIG-SCIG | 9 | -0.55 | 0.13 |
| SCIG-follow-up | 6 | 0.58 | 0.23 |

Correlation analyses were made with Spearman correlation coefficient in six of seven comparisons.

For IVIG (IVIG-SCIG study) Pearson correlation coefficient was used due to Gaussian distribution)
